# A88 EVALUATING THE ACCEPTABILITY AND EFFICACY OF CYTOSPONGE FOR BARRETT'S ESOPHAGUS: A SINGLE CENTRE CROSS-SECTIONAL STUDY

**DOI:** 10.1093/jcag/gwac036.088

**Published:** 2023-03-07

**Authors:** A E Alqattan, L Calman T'ien, M Choi, B Chan, C E Galorport, R A Enns

**Affiliations:** Internal Medicine, Division of Gastroenterology, UBC, St. Paul's Hospital, Vancouver, BC, Canada

## Abstract

**Background:**

Barrett's Esophagus (BE) is a pre-malignant condition defined by the presence of metaplastic columnar epithelial cells above the gastroesophageal junction. Currently, diagnoses is made by endoscopy. Once metaplasia is present, there is 0.5% annual risk of progression to dysplasia and ultimately adenocarcinoma.

Cytosponge is a new device and technique to diagnose BE. Furthermore, this test has a strong safety profile. Research has suggested increased patient tolerance for the Cytosponge compared to endoscopy; this has not been demonstrated in a Canadian healthcare setting. Before Cytosponge test can be integrated in Canada, ideally, patient acceptability of this device should be evaluated.

**Purpose:**

To assess patient acceptability, tolerability and integration of Cytosponge in the diagnosis of Barrett's Esophagus in a Canadian healthcare setting. We also assessed the ease of use and familiarity with Cytosponge.

**Method:**

A single-centre, prospective cross-sectional study was conducted to evaluate the acceptability and comfort of patients undergoing Cytosponge procedure.

Outpatients referred for EGD for Barrett's Esophagus at St. Paul’s Hospital between 03/21-07/22 were included. 36 patients with BE have been enrolled in this project. Acceptability was evaluated through Visual Analogue Scale (VAS), Spielberger State Trait Anxiety Inventory (STAI), and Impact of Events Scale (IOES) on the day of procedure, day 7 post procedure and day 90 post procedure. Data from health care providers administering the Cytosponge were collected using the System Usability Scale (SUS).

One-way ANOVA and Tukey’s Honestly Significant Difference tests were completed to assess score differences between follow up.

**Result(s):**

A total of 36 patients met the inclusion criteria and consented to participate. Of these patients 81.6% were successful in swallowing Cytosponge, 18.4% were unsucessful. ANOVA test revealed statistically significant difference in VAS scores, F(3, 140) = 12.59, p < 0.0000005. There were significant differences in VAS between Day 0 and Day 7, p=0.0032. This was also seen in VAS between Day 0 vs Day 90, p=0.0017.

There were no statistically significant difference in mean STAI scores between different time points, F(3, 140) = 12.59, p=0.44. ANOVA test also showed statistical difference in IOES scores, F(2, 111) = 8.76, p<0.0005. There was statistical difference between day 0 compared to day 7 and between day 0 and day 90, p=0.0045, and p=0.00045 respectively.

**Conclusion(s):**

Our results demonstrate that Cytosponge is a well tolerated in a Canadian healthcare setting. Follow up scores of VAS and IOES were lower compared to day 0 suggesting that patients found Cytosponge acceptable. A score of 68 and above is considered to be above average on the SUS which measures usability of Cytosponge. The average SUS score in this sample was 65.3, this may suggest that there is a learning curve for health care providers to become familiar with Cytosponge. There were no complications with Cytosponge in this sample.

**Please acknowledge all funding agencies by checking the applicable boxes below:**

Other

**Please indicate your source of funding;:**

Gastroenterology Institute of Research Institute

**Disclosure of Interest:**

None Declared

